# Autophagy and PXR Crosstalk in the Regulation of Cancer Drug Metabolism and Resistance According to Gene Mutational Status in Colorectal Cancer

**DOI:** 10.3390/genes16080892

**Published:** 2025-07-28

**Authors:** Evangelos Koustas, Panagiotis Sarantis, Eleni-Myrto Trifylli, Eleftheria Dikoglou-Tzanetatou, Evangelia Ioakeimidou, Ioanna A. Anastasiou, Michalis V. Karamouzis, Stamatios Theocharis

**Affiliations:** 1 Oncology Department, General Hospital Evangelismos, Ipsilantou 45-47, 106 76 Athens, Greece; 2 First Department of Pathology, Medical School, National and Kapodistrian University of Athens, 115 27 Athens, Greece; panayotissarantis@gmail.com (P.S.); eltzanetatou@yahoo.com (E.D.-T.); ioak.evelina@gmail.com (E.I.); statheocharis@yahoo.com (S.T.); 3 University Pathology Clinic, General and Oncology Hospital “Agioi Anargyroi”, National and Kapoditrian University of Athens, Timiou Stavrou 14, 145 64 Kifisia, Greece; mkaramouz@med.uoa.gr; 4 GI-Liver Unit, 2nd Department of Internal Medicine, National and Kapodistrian University of Athens, General Hospital of Athens “Hippocratio”, 114 Vas Sofias, 115 27 Athens, Greece; trif.lena@gmail.com; 5 Diabetes Center, First Department of Propaedeutic Internal Medicine, Medical School, National and Kapodistrian University of Athens, Laiko General Hospital, 115 27 Athens, Greece; anastasiouiwanna@gmail.com; 6 Department of Pharmacology, Medical School, National and Kapodistrian University of Athens, 115 27 Athens, Greece

**Keywords:** autophagy, colorectal cancer, irinotecan, pregnane X receptor—PXR

## Abstract

**Background and Objectives:** Colorectal cancer (CRC) is one of the most frequently diagnosed malignancies worldwide. Although chemotherapy is an effective treatment for colorectal cancer (CRC), its effectiveness is frequently hindered by the emergence of resistant cancer cells. Studies have demonstrated a linkage between drug resistance and the pregnane X receptor (PXR), which influences the metabolism and the transport of chemotherapeutic agents. Likewise, autophagy is also a well-established mechanism that contributes to chemotherapy resistance, and it is closely tied to tumor progression. This pre-clinical study aims to investigate the role of mtKRAS-dependent autophagy with PXR expression after treatment with Irinotecan in colorectal cancer. **Methods:** CRC lines were treated with specific inhibitors, such as 3-methyladeninee, hydroxychloroquine PI-103, and irinotecan hydrochloride, and subjected to various assays, including MTT for cell viability, Western blot for protein expression, siRNA-mediated PXR knock-out, and confocal microscopy for autophagic vacuole visualization. Protein quantification, gene knockdown, and subcellular localization studies were performed under standardized conditions to investigate treatment effects on autophagy and apoptosis pathways. **Conclusions:** Our experiments showed that PXR knockdown does not alter autophagy levels following Irinotecan treatment, but it promotes apoptotic cell death despite elevated autophagy. Moreover, late-stage autophagy inhibition reduces PXR expression, whereas induction through PI3K/AKT/mTOR inhibition leads to increased expression of PXR. Our experiments uncover a mechanism by which autophagy facilitates the nuclear translocation of the PXR, thereby promoting resistance to Irinotecan across multiple cell lines.

## 1. Introduction

Colorectal cancer (CRC) constitutes the third most commonly diagnosed malignancy, and the second deadliest, among all malignancies worldwide, with more than 1.9 million new cases and almost 1 million deaths in 2020, respectively. As global life expectancy increases, more people are reaching ages at which colorectal cancer (CRC) is more likely to develop. However, aging is not the only cause, as unhealthy lifestyles and urbanization also increase the risk for CRC in many developing countries. Meanwhile, it has to be underlined that more and more CRC cases are identified, a phenomenon that is mainly attributed to improved CRC screening programs, as well as to the widespread use of colonoscopy and other diagnostic and screening tools, which may detect more cases, even at earlier stages [1]. Despite the availability of several chemotherapeutic agents for CRC, the treatment response is often limited due to the development of resistant neoplastic cells. The development of novel therapeutic targets, as well as the identification and management of potential drug resistance mechanisms, are considered essential for this highly common malignancy, which presents metastatic dissemination in almost 20% of the cases [2].

Autophagy is a highly regulated catabolic mechanism that has a pivotal role in colorectal carcinogenesis. The orchestration of this catabolic process is mediated by autophagy-related genes, which express various proteins, such as Beclin-1, MAP1LC3B, and p62/SQSTM1, that not only regulate this multi-step procedure but also are closely associated with carcinogenesis, including CRC [3]. More particularly, this homeostatic and energy-conserving cellular function is also implicated in migration, angiogenesis, as well as in various cell functions, such as proliferation and epithelial-mesenchymal transition [3,4], demonstrating a dual role for CRC. Additionally, the autophagy pathway is activated under conditions such as hypoxia, which can manifest in tumor regions, where high-grade malignant tumor cells are established, as a result of their increased oxygen demands for proliferation and expansion [5]. Meanwhile, it has to be noted that autophagy induction during chemotherapy has led to chemoresistance and reduced survival rates in CRC patients [6].

Pregnane X receptor (PXR) constitutes a key regulator for a wide variety of target genes, while it also participates in many physiological and non-physiological conditions through multiple cellular circuits, such as oxidative stress, inflammation, and apoptosis. At the same time, it is also considered a regulator for the cell cycle, tissue growth, and biotransformation [7,8]. Furthermore, PXR is associated with liver regeneration and hepatic proliferation, implying its participation in tumor progression, angiogenesis, and metastatic dissemination [9]. Meanwhile, PXR is not only implicated in carcinogenesis and tumor progression, but also in multi-drug resistance and inefficient chemotherapy response. On top of that, the impact of PXR is also reported in many hematological malignancies and various cancers of the gastrointestinal system, such as liver, esophagus, colon, and pancreas cancer, as well as in the breast, endometrium, cervix, ovaries, lung, and prostate. Additionally, PXR overexpression and altered subcellular localization resulting from mutations have been associated with endometrial, breast, and colorectal cancers [6]. Elevated PXR mRNA levels have also been observed in esophageal adenocarcinoma and Barrett’s epithelium, whereas PXR protein was undetectable in normal esophageal epithelium but was present in the nuclei of malignant cells [10,11]. Moreover, PXR mutation leads to alterations in subcellular position and upregulated expression of several genes that lead to CRC development, similar to breast and endometrial malignancy. On the other hand, PXR has a crucial role in detoxification and homeostatic mechanisms [7], implying its dual role, either as a tumor promoter or inhibitor, as well as its potential utilization as a target for anti-neoplastic therapeutic strategies.

PXR is a key regulator of drug-metabolizing enzymes (DMEs), a diverse group of proteins crucial for transforming and eliminating both endogenous compounds and foreign substances like drugs, carcinogens, and environmental pollutants from the body (like CYP3A4) and drug transporters (like MDR1), which are responsible for the breakdown and removal of drugs from the body. Overexpression of PXR can lead to increased levels of these proteins, resulting in faster drug metabolism and efflux, thus reducing the concentration of therapeutic agents within cells and contributing to drug resistance. Furthermore, PXR has a major role in the expression of multiple DMEs of phase I and phase II, including uridine diphosphate (UDP)-glucuronosyltransferases, cytochrome P450, carboxylesterase [12], as well as in subcellular transport. Its role in the latter function was also demonstrated in a study by, in which it was reported that PXR could be utilized as a biomarker for the prediction of resistance to platinum agents or oxaliplatin (L-OHP) transport capacity (L-OHP) in CRC [13]. At the same time, it regulates the function of ATP-binding cassette transporter C 2 (ABCC), drug-efflux pumps multidrug resistance gene 1 (*MDR1*), *MDR2*, and anion-transporting polypeptide 2 (OATP) [13]. Meanwhile, CYP3A4 constitutes an enzyme that metabolizes the majority of drugs (more than 50% of them), including many chemotherapeutic agents. The aforementioned enzyme is closely regulated by PXR, resulting in chemoresistance and limited response to anticancer treatment, as well as to dismal prognosis. Moreover, studies have shown that UDP-glucuronosyltransferases (UGTs) such as UGT1A9 and UGT2B7 can interact with and suppress CYP3A4 activity despite previously being thought to function independently [10]. Nevertheless, several studies demonstrate that PXR promotes tumor sensitivity to chemotherapeutic agents by regulating the gene expression of drug transporters and via its implication in several metabolic processes that influence the treatment outcome of many antineoplastic agents, including taxanes, platinum-based agents, and antihormone agents (such as paclitaxel, cisplatin, tamoxifen), as well as topoisomarase-I inhibitors such as doxorubicin and ixabepilone [13,14]. Ultimately, it has to be emphasized that the interplay between CRC cellular resistance and the PXR nuclear factor minimizes the treatment response to Irinotecan, which is a DNA topoisomerase I inhibitor class of anti-neoplastic drugs for several solid tumors, including CRC. Notably, autophagy induction during chemotherapy promotes chemoresistance and reduces survival in CRC patients. Based on the aforementioned, further investigation is needed to establish the interplay between the autophagy pathway and expression in many malignancies, demonstrating a dual role in carcinogenesis, either as a tumor stimulator or suppressor [15,16]. This pre-clinical study aims to shed light on assessing the impact of autophagy on PXR regulation in CRC cell lines and the role of mtKRAS-dependent autophagy with PXR expression after treatment with Irinotecan in colorectal cancer.

## 2. Materials and Methods

### 2.1. Inhibitors and Drugs

3-Methyladeninee (3-MA), (#13242 Caymanchemical Company, Ann Arbor, MI, USA), Hydroxychloroquine (HCQ) (#H0915 Sigma-Aldrich, St. Louis, MO, USA), PI-103 (#S1038 Selleckchem, Houston, TX, USA), and Irinotecan hydrochloride (#1347609 Merck, Darmstadt, Germany) were used.

### 2.2. Cell Lines

HT29 (HTB-38), Colo-205 (CCL-222), RKO (CRL-2577), HCT116 (CCL-247), DLD-1 (CCL-221), and SW-480 (CCL-228) human colon adenocarcinoma cell lines were obtained from the American Type Culture Collection (ATCC, Manassas, VA, USA). All CRC cell lines were grown in Dulbecco’s Modified Eagle Medium supplemented with 10% fetal bovine serum, L-glutamine, vitamins, penicillin, streptomycin antibiotics, and amino acids (Invitrogen, Carlsbad, CA, USA). Cells were incubated at 37 °C in a humidified incubator containing 5% CO_2_. The experiments were performed with the approval of the Ethics Committee of our university.

### 2.3. Cell Viability Assay

Cell growth and viability were assessed using an MTT assay. About 3000 cells were seeded in a 96-well plate with 200 μL of culture medium. Each experiment was performed in triplicate. After the treatment, the cells were incubated for 4 h with 0.8 mg/mL of MTT, dissolved in serum-free medium, and followed by the addition of 1 mL of DMSO. The mixture was gently shaken for 10 min to ensure complete dissolution. Lastly, absorbance readings were obtained at 560 nm using a microplate spectrophotometer (SpectraMax 190-Molecular Devices, Molecular Devices, San Jose, CA, USA). The results are expressed as a percentage of the control values.

### 2.4. Western Blot Assay

As previously described [17], whole-cell lysates were prepared using RIPA buffer (#9806 Cell Signaling Technology, Danvers, CA, USA). A total of 25 μg of protein, quantified via the Bradford method, was separated using SDS-PAGE and transferred to a nitrocellulose membrane (Whatman, Scheicher & Schuell, Dassel, Germany). The following antibodies were used: LC3B (D11) #3868, SQSTM1/p62 #8025, cleaved caspase-3 #9661, and PARP-1 #9542, all obtained from Cell Signaling Technology. Additionally, antibodies against PXR (G-11, sc-48403) and actin (sc-8035) were sourced from Santa Cruz Biotechnology. All antibodies were diluted at a ratio of 1:1000, as specified in the datasheet for each respective antibody. The antibody signal was detected using an enhanced chemiluminescence and specific detection system (Amersham Biosciences, Uppsala, Sweden) following exposure to Fuji medical X-ray film. These results are derived from three separate experiments, and the standard deviation is indicated. Protein levels were normalized against actin, and ImageJ 1.41 was used to measure the intensities of the protein bands.

### 2.5. siRNA and Transfection

For the siRNA transfection experiments, we used siRNA to knock down PXR (sc-44057, Santa Cruz Biochemicals, Santa Cruz, CA, USA) and Lipofectamine 3000 (#L3000-15 Invitrogen Corp.). According to the manufacturer’s instructions, the mtKRAS CRC cell lines, DLD-1, HCT116, and SW480, were exposed to 80 nM siRNA-PXR for 48 h according to the manufacturer’s recommendations.

### 2.6. Two-Dimensional Culture and Confocal Microscopy

For the 2D culture experiments, the cells (5000 cells/well) were grown on coverslips in 24-well plates in culture medium at 37 °C. CRC cell lines were treated with 10 μΜ of Irinotecan, 10 mM of HCQ, and 1μΜ of dual inhibitor against AKT/mTOR, PI-103, alone or in combination, for 48 h. Cells were fixed with 4% paraformaldehyde, washed with PBS, and immediately analyzed using confocal microscopy to detect the autophagic vacuoles. MDC was used as an auto-fluorescent marker (512 nm) that is preferentially gathered in autophagic vacuoles, with treatments applied to denote incubation times. The accumulation of MDC in autophagic vacuoles results from ion trapping and specific interactions with the lipids of the vacuole membrane. Additionally, the cell cytoskeleton was stained using phalloidin (Alexa Fluor 546, A22283, Life technologies, Carlsbad, CA, USA). Subcellular localization of PXR was detected with the primary PXR antibody and then incubated with an anti-mouse fluorescence-labeled secondary antibody (Alexa Fluor 488, A28175, Life technologies). Finally, the cells were examined using an Olympus FV1000 confocal microscope with a digital camera.

### 2.7. Statistical Analysis

The results are representative from at least three independent experiments and expressed as mean values ± SD (standard deviation). The results were evaluated by TTEST. Statistical significance was inferred when *p* < 0.05.

## 3. Results

### 3.1. Irinotecan-Dependent Autophagy Induction as a Protective Mechanism—The Role of PXR in Irinotecan Treatment

**Figure 1:**
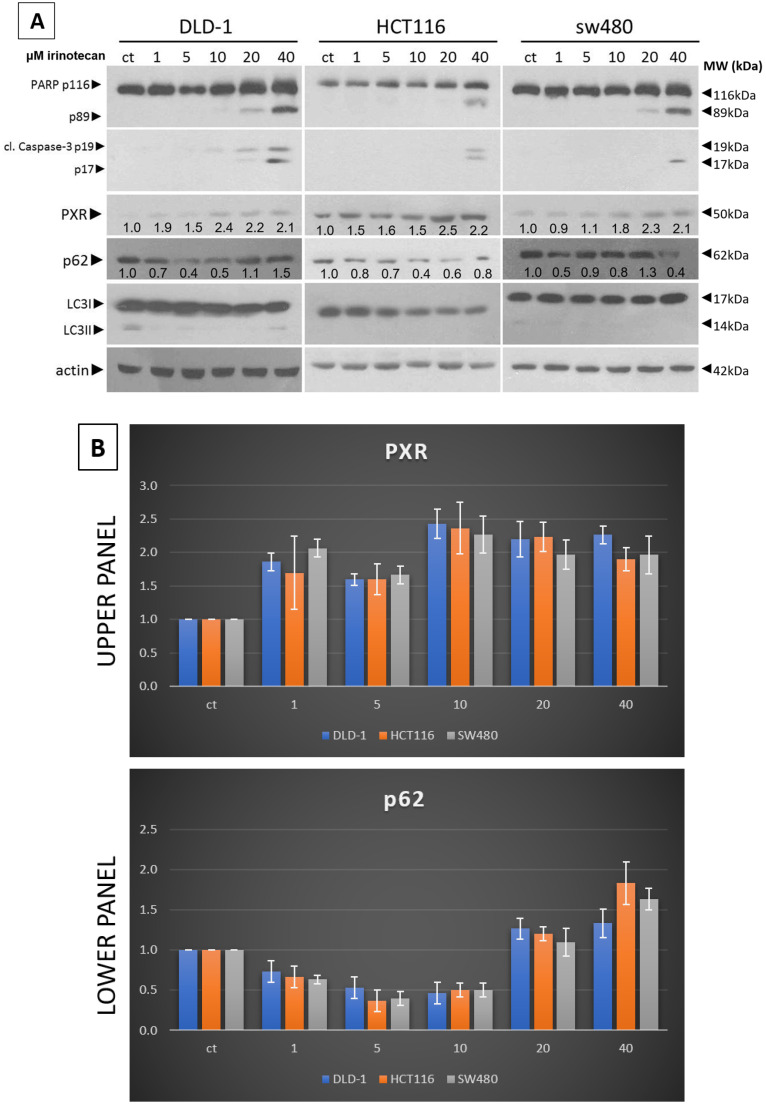
Irinotecan enhances the expression of PXR and autophagy in mtKRAS CRC cell lines. (**A**) Western blot analysis after 48 h exposure of mutant KRAS CRC cell lines (DLD-1, HCT116, and SW480) in 1, 5, 10, 20, and 40 μΜ of Irinotecan. Apoptotic cell death was determined by as-sessing the levels of cleaved PARP and caspase-3 proteins. Additionally, the protein levels of PXR and autophagy markers LC3 and p62 are also reported. These protein levels were normalized against actin. (**B**) Protein levels of PXR (upper panel) and p62 (lower panel), normalized to actin, in three different mtKRAS cell lines treated with varying concentrations of irinotecan.

It is reported that Irinotecan causes growth inhibition and apoptosis in tumor cells; to confirm this phenomenon, CRC cell lines, DLD-1, HCT116, and SW480 cells were treated with Irinotecan at various concentrations (1, 5, 10, 20, and 40 μΜ) for 48 h. Two important apoptosis-related signaling molecules, (i) cleaved caspase 3 and (ii) cleaved PARP, were analyzed next. As is shown in Figure 1, the expression of cleaved PARP and cleaved caspase-3 increased in a dose-dependent manner, suggesting that the apoptosis in all three mtKRAS CRC cells was induced by Irinotecan. PXR protein levels and the autophagy markers LC3 and p62 were also further detected (Figure 1). In all three mtKRAS CRC cells, the PXR protein levels were increased (1.1- to 2.4-fold) after treatment with an increasing dose of Irinotecan. An increase in PXR can significantly alter the expression of target genes involved in drug metabolism, efflux, and detoxification. This effect may plateau due to feedback inhibition or cytotoxicity at excessive doses of irinotecan. In DLD-1 cell lines, 1, 5, and 10 μΜ of Irinotecan triggered autophagy, which was identified through p62 and LC3 (decreasing and increasing, respectively) protein levels through Western blot analysis. In a second mtKRAS cell line, HCT116, autophagy was initiated, as confirmed by the levels of p62 and LC3II, in the same manner as DLD-1, but with a stronger reduction in p62, in all doses of Irinotecan. In the SW480 cell line, strong autophagy induction was detected with 1 and 40 μΜ of Irinotecan; this was identified through p62 and LC3 (decreasing and increasing, respectively) protein levels (Figure 1). These results indicated that Irinotecan triggers autophagy, PXR, and apoptosis in a dose-dependent manner in CRC mtKRAS cell lines.

### 3.2. Silence of PXR Triggers Apoptotic Cell Death After Treatment with Irinotecan

**Figure 2:**
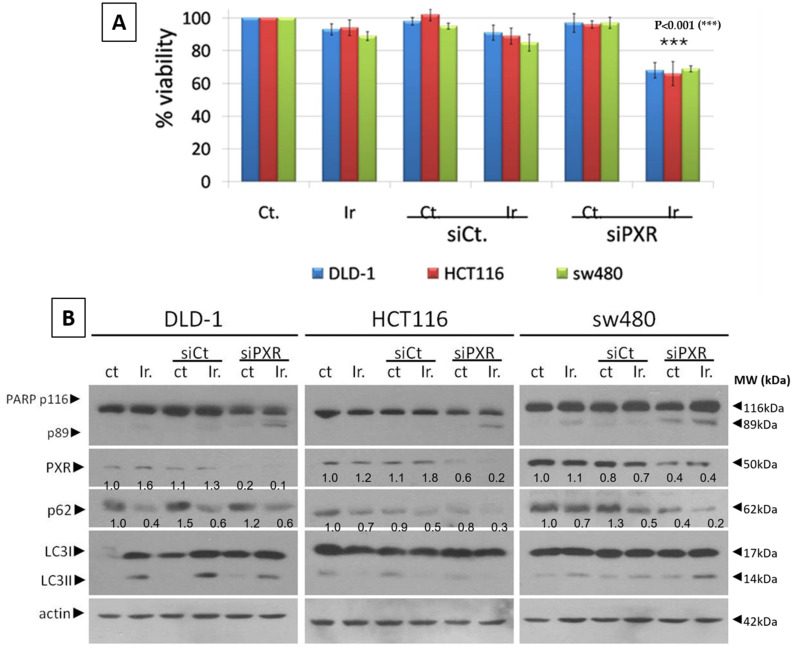

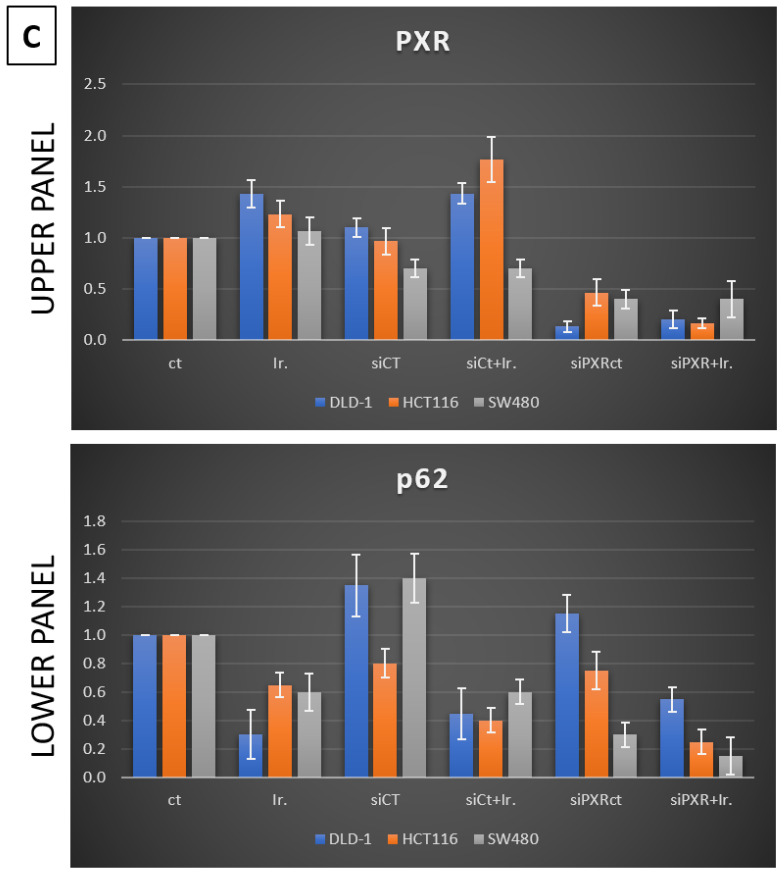
Silence of PXR triggers apoptotic cell death and reduces cell viability after treatment of mtKRAS cell lines with Irinotecan. (**A**) The viability of the mutant KRAS CRC cell lines DLD-1, HCT116, and SW480 was examined with the MTT viability assay. Cell lines were exposed to 10μΜ of Irinotecan alone and 10μΜ Irinotecan after silencing of PXR expression with 80 nM siRNA for 24 h. Also, control siRNA transfection was carried out under the same conditions. In the case of siPXR, there is statistical significance between the control and the incubation with Irinotecan across all three cell lines. In siPXR plus irinotecan *p* < 0.001 (***) (**B**) Western blot analysis after 48 h the exposure of mutant KRAS CRC cell lines DLD-1, HCT116, and SW480 to 10μΜ Irinotecan, and with 10μΜ Irinotecan after silence of PXR expression with 80 nM siRNA. Apoptotic cell death was identified through cleaved PARP and caspase-3 protein levels. The protein levels of PXR and autophagy markers LC3 and p62 are also presented. Protein levels were normalized against actin. (**C**) Protein levels of PXR (upper panel) and p62 (lower panel), normalized to actin, in three different mtKRAS cell lines treated with 10μΜ concentrations of irinotecan after silencing of PXR expression with 80 nM siRNA for 24 h.

Due to the activation of autophagy in mtKRAS CRC cell lines (HCT116, DLD-1, and SW480) after being treated with Irinotecan and to explore the mechanisms by which both PXR expression and autophagy are increased and activated, we treated all three mtKRAS cell lines (HCT116, DLD-1, and SW480) with 10 μΜ of Irinotecan after silencing PXR expression with 80 nM of siRNA. The concentration of 10 μM of Irinotecan was chosen for the experiments involving PXR siRNA based on its demonstrated effect on PXR protein expression and the stronger induction of autophagy according to p62 protein levels. The response of HCT116, DLD-1, and SW480 CRC cell lines was measured after treatment with 10 μΜ of Irinotecan and/or siRNA against PXR for 48 h (Figure 2A) by using the MTT-viability assay. Based on our observations, cell viability of all three cell lines was reduced by around 10% after treatment with Irinotecan alone, whereas it was reduced by 10–15% after the successful knockdown of PXR (as identified through Western blot analysis—Figure 2B upper panel), compared to control. Subsequently, due to the induction of PXR expression after the treatment of mtKRAS cell lines with Irinotecan, we treated all three mtKRAS cells with 10μΜ of Irinotecan after the knockdown of PXR expression with siRNA. In all three cell lines, a further reduction in cell viability of 50–55% was observed after 48 h (Figure 2A). The observed differences are statistically significant for each cell line, with *p*-values of 0.012 (DLD-1), 0.005 (HCT116), and 0.002 (sw480).

We further investigated the reduction of mtKRAS CRC cell lines (HCT116, DLD-1, and SW480) viability and we showed that PXR deficiency. Apoptotic cell death was confirmed via Western blot analysis following siRNA treatment targeting PXR, which induced cell apoptosis in all three cell lines, as evidenced by the detection of cleaved PARP-1 after their exposure to 10 μM Irinotecan (Figure 2B). Moreover, autophagy was activated at all Irinotecan treatment points, indicated by the decrease in p62 levels and the increase in LC3 protein levels (Figure 2B). Figure 2C displays representative immunoblots of PXR (upper panel) and p62 (lower panel), with actin used as a loading control. Corresponding bar charts present the quantified protein levels for each mtKRAS cell line at 20μΜ irinotecan concentrations after silencing of PXR. The graphs highlight relative expression changes across treatments, allowing for direct comparison. This combined presentation facilitates clearer insight into the protein expression profiles (Figure 2C).

### 3.3. Autophagy Regulates the Expression of PXR in mtKRAS CRC Cell Lines

**Figure 3:**
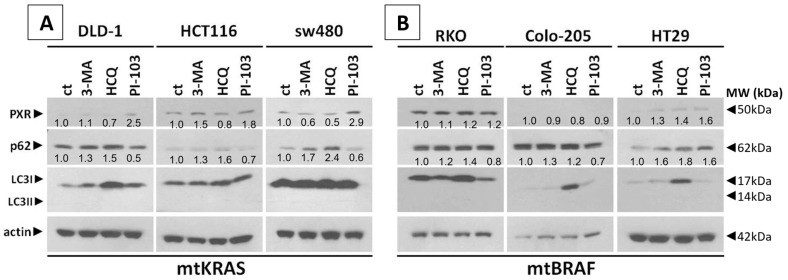

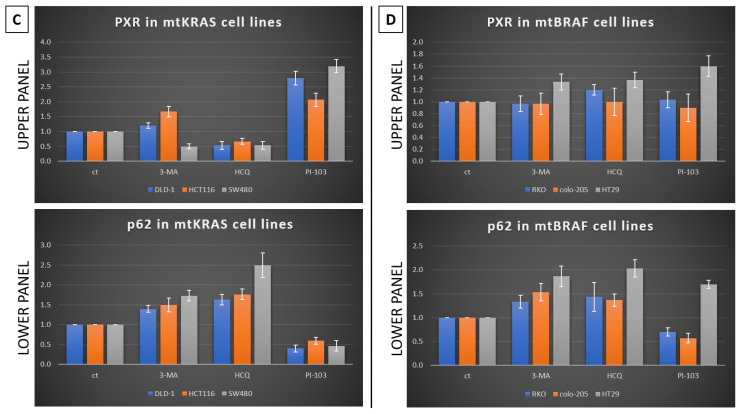
Autophagy regulates the expression of PXR. Western blot analysis after 24-h exposure of three mutant KRAS (**A**) and three mutant BRAF (**B**) CRC cell lines DLD-1, HCT116, SW480(DLD-1, HCT116, SW480, and RKO, colo-205, HT29, respectively) were exposed totwo autophagy inhibitors, 5mM 3-MA, 10mM HCQ, and a dual inhibitor of AKT/mTOR, 1μΜ PI-103, for 24 h. The protein levels of PXR and the autophagy markers p62 and LC3B were measured through western blot analysis. Protein levels were normalized against actin. (**C**,**D**) Protein levels of PXR (upper panel) and p62 (lower panel), normalized to actin, in three different mtKRAS and mtBRAF CRC cell lines treated with three different agents. Bar graphs alongside the blots depict the quantified protein levels in each cell line across all three different agents.

Aiming to identify the effect of autophagy on PXR expression, three mtKRAS and three mtBRAF CRC cell lines (DLD-1, HCT116, and SW480 and RKO, colo-205, and HT29, respectively) were exposed to two autophagy inhibitors (5 mM of 3-MA and 10 mM of HCQ) and a dual inhibitor of AKT/mTOR (1μΜ of PI-103) for 24 h. It is well established that KRAS and BRAF regulate two key signaling pathways, KRAS–BRAF–MEK–ERK and KRAS–PI3K–AKT–mTOR, both of which have been implicated in the regulation of autophagy. Therefore, we employed three cell lines harboring mutant KRAS and three with mutant BRAF to investigate the impact of these mutations and the consequent overactivation of their associated pathways on autophagy and PXR. Moreover, these two signaling pathways appear to exert opposing effects on autophagy: while the BRAF–MEK–ERK pathway promotes autophagy, the PI3K–AKT–mTOR pathway inhibits it. The protein levels of PXR and the autophagy markers p62 and LC3B were measured through Western blot analysis. In all three mtKRAS CRC cell lines, the protein levels of PXR were significantly decreased (0.5- to 0.8-fold) after their exposure to HCQ. Additionally, the protein levels of PXR did not significantly change after treatment with 3-MA, except in the SW480 cell line, where the reduction was 0.6-fold (Figure 3A). In contrast to the utilization of autophagy inhibitors, when the mtKRAS CRC cell lines were treated with a dual inhibitor for AKT/mTOR, PI-103, the protein levels of PXR were significantly elevated (1.8- to 2.9-fold). Moreover, autophagy initiation was identified by the protein levels of p62 and LC3. In both autophagy inhibitors (3-MA and HCQ), p62 (1.2- to 2.4-fold) and LC3 were increased. Meanwhile, the inhibition of the AKT/mTOR signaling pathway (a well-known autophagy modulator) led to a decrease and increase in p62 (0.5- to 0.7-fold) and LC3, respectively (Figure 3A).

Furthermore, in two mtBRAF CRC cell lines (RKO and colo-205) in the same condition, the protein levels of PXR did not significantly change (0.8- to 1.2-fold) after the inhibition or induction of autophagy. In the HT29 CRC cell line, the protein levels of PXR were elevated in all conditions. In addition, the same cell line autophagy did not activate after inhibition of AKT/mTOR, as was identified through the protein levels of p62 and LC3 (Figure 3B).

Figure 3C,D shows representative immunoblots of PXR (upper panel) and p62 (lower panel), with actin serving as the loading control in mtKRAS and mtBRAF CRC cell lines. Bar graphs alongside the blots depict the quantified protein levels in each cell line across all three different agents. These visual data emphasize treatment-related changes in expression, enabling comparative analysis. Together, the blots and graphs provide a comprehensive view of protein PXR and p62 expression dynamics (Figure 3C,D).

Based on our experiments, autophagy modulation appears to alter the expression of PXR in mtKRAS cell lines.

### 3.4. Autophagy Regulates the Subcellular Localization of PXR in mtKRAS CRC Cell Lines

Further analysis of autophagy’s role in PXR expression required the investigation of the subcellular localization of PXR after manipulation of the autophagy process.

**Figure 4:**
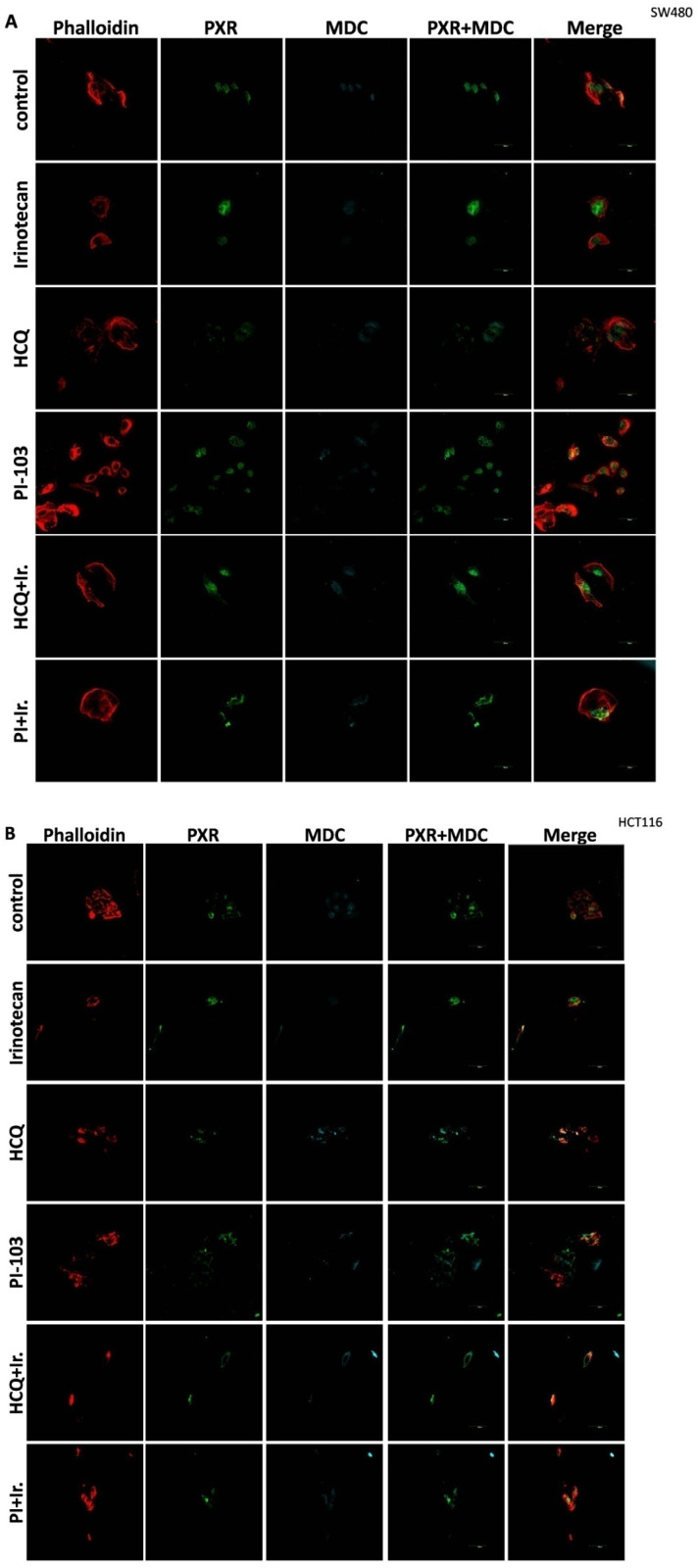

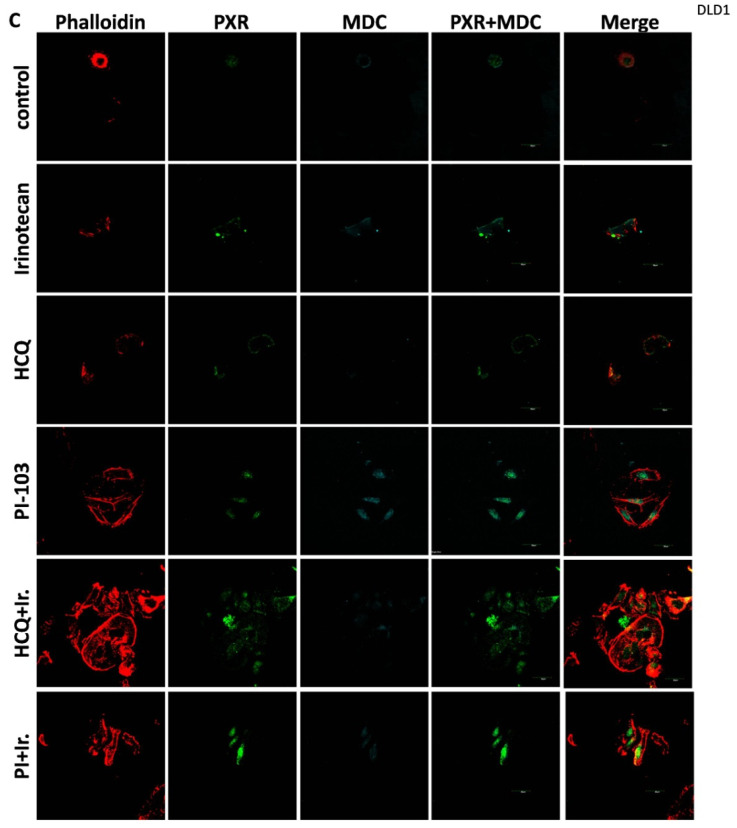
**Autophagy regulates the subcellular localization of PXR.** All three mtKRAS cell lines, SW480 (**A**), HCT116 (**B**), and DLD-1 (**C**), were treated with 10 μM Irinotecan, 10 mM HCQ, and 1 μM of the dual inhibitor against AKT/mTOR, PI-103, for 48 h. To further confirm the expression of autophagy, MDC staining (light blue) showed the presence of autophagic vacuoles in a significant proportion of cells stained with phalloidin (red). The sub-cellular localization of PXR was revealed with a specific antibody against this protein (green).

All three mtKRAS cell lines (HCT116, DLD-1, and SW480) were subjected to treatment with 10 μM of Irinotecan, 10 mM of HCQ, and 1 μM of the dual AKT/mTOR inhibitor, PI-103, for the duration of 48 h. To further confirm the expression of autophagy, MDC staining (light blue) was performed, which showed a significant presence of autophagic vacuoles in a large percentage of the phalloidin-stained cells (red). Additionally, the subcellular localization of PXR was revealed using a specific antibody targeting this protein (green) (Figure 4A–C).

As was identified through Western blot analysis (Figure 2), Irinotecan activates autophagy in all three mtKRAS CRC cell lines. Autophagy activation was confirmed through MDC staining with confocal microscopy in all three mtKRAS CRC cell lines. In addition, increased PXR (green) levels were detected in all three mtKRAS cell lines in the cytoplasm (Figure 4A–C).

Inhibition of autophagy with hydroxychloroquine (HCQ) decreased the subcellular localization of PXR in the cytoplasm in all three mtKRAS cell lines. Induction of autophagy with a dual inhibitor of AKT and mTOR (PI-103) increased the protein levels of PXR (green) and the number of autophagic vacuoles (MDC staining-light blue). Furthermore, after the induction of autophagy with PI-103, the subcellular localization of PXR was altered from cytoplasm to the nucleus in all three mtKRAS cell lines (Figure 4A–C). Co-treatment of HCT116, DLD-1, and SW480 with HCQ + Irinotecan and PI-103 + Irinotecan did not affect the subcellular localization of PXR (in the cytoplasm) as was mentioned with HCQ and PI-103 treatment alone (Figure 4A–C).

Thus, autophagy induction increases the subcellular localization of PXR to the nucleus. On the other hand, autophagy inhibition with HCQ reduced the transport of PXR into the nucleus and, consequently, its accumulation in the cytoplasm.

## 4. Discussion

This study investigates the role of mtKRAS-dependent autophagy with PXR expression after treatment with Irinotecan in CRC. The data indicate that autophagy leads to PXR expression in the mtKRAS CRC cell line as a protective mechanism against Irinotecan in colorectal cancer cell lines. Our findings show that Irinotecan induces autophagy, which, in turn, increases PXR expression. In addition, PXR exerts antiapoptotic effects in mtKRAS CRC cell lines. Consequently, inhibition of autophagy may lead not only to a reduction in overall PXR levels but also to the accumulation of residual PXR in the cytoplasm rather than its translocation to the nucleus.

Autophagy is a controversial mechanism involved in both the survival and growth suppression of tumor cells [18,19,20]. In this study, we have examined the effect of Irinotecan on the induction of autophagy and PXR expression in mtKRAS CRC. KRAS is a small GTPase that plays a central role in regulating cell proliferation, differentiation, and survival through key signaling pathways such as MAPK/ERK and PI3K/AKT. When it is mutated (commonly referred to as mtKRAS), it becomes constitutively active, driving oncogenic signaling that contributes to tumorigenesis and resistance to therapy. These mutations are particularly prevalent in pancreatic, colorectal, and lung cancers, and they are associated with poor prognosis. mtKRAS reprograms cellular metabolism to support rapid tumor growth, enhances glycolysis and glutaminolysis, and promotes immune evasion. Moreover, mtKRAS influences the tumor microenvironment and may interact with autophagy and nuclear receptor trafficking, which further suggest its complex role in chemoresistance. Understanding the distinct biological behavior of mtKRAS compared to wild-type KRAS is essential for developing targeted therapies and improving clinical outcomes in KRAS-driven cancers. The inconsistent regulation of PXR observed in mtBRAF cell lines, such as HT29, compared to mtKRAS lines may reflect differences in downstream signaling pathway activation. BRAF mutations predominantly activate the MAPK/ERK pathway, often leading to sustained and focused transcriptional changes. In contrast, KRAS mutations can activate multiple pathways, including PI3K/AKT, which may exert distinct modulatory effects on nuclear receptors such as PXR. This divergence in pathway engagement could explain the heterogeneous expression profiles, with HT29 cells potentially favoring MAPK-dependent transcriptional programs that do not consistently regulate PXR.

PXR is a nuclear receptor that influences drug metabolism, drug efflux, and drug–drug interactions by regulating multiple drug resistance 1 (*MDR1*), indicating its critical role in multidrug resistance and other related genes [21]. Autophagy is characterized by the formation of the autophagosome, a double-membrane structure that is closely associated with the LC3B protein. In numerous solid tumors, such as colorectal cancer (CRC), LC3B staining is typically indicative of elevated autophagy levels. Numerous studies have established that autophagy serves as a cytoprotective mechanism in various types of cancer [22]. Our experiments show that a low dose of Irinotecan enhances autophagy in the mtKRAS CRC cell line. Initiation of the process of autophagy was detected through reduced and increased levels of p62 and LC3B, respectively [23].

Furthermore, in the same cell lines, Irinotecan increased the expression of PXR. At high doses, Irinotecan inhibited autophagy in a manner similar to HCQ, a well-known late-stage autophagy inhibitor, and consequently triggered apoptotic cell death. The inhibition of autophagy was confirmed by the accumulation of both p62 and LC3B. This observation provides the first evidence of a correlation between the protective role of autophagy and PXR expression.

Meanwhile, recent studies highlight that the activation of PXR also plays a crucial role in regulating apoptosis and in acquired resistance to chemotherapeutic agents [24], while others have identified the role of autophagy as a cytoprotective mechanism in several cancer types, including CRC [23]. Based on our experiments, PXR expression is dependent on the autophagy mechanism. As was identified through our experiments, the knockdown of PXR did not affect autophagy levels after treatment with Irinotecan. Moreover, the lack of PXR expression leads to apoptotic cell death due to Irinotecan treatment, despite high levels of autophagy, demonstrating the positive effect of autophagy in PXR expression. mtKRAS CRC cell lines were treated with autophagy inhibitors 3-MA and HCQ and autophagy inducer PI-103 [25]. Inhibition of autophagy in the later stage of the process (maturation to fusion with lysosome) leads to a reduction in PXR expression. On the other hand, induction of autophagy through PI3K/AKT/mTOR inhibition leads to increased expression of PXR [26]. Thus, we conclude that autophagy is the response mechanism that controls the expression of PXR in CRC cell lines. Meanwhile, the reduction in PXR after inhibition of autophagy in the maturation/autophagosome–lysosome fusion stage with HCQ is the first indication of endosomal PXR transport alteration [27]. It is well known that PXR translocates from the cytoplasm to the nucleus of cells when it is bound to and activated by ligands. Then, it binds to its DNA response elements as a heterodimer or heterotetramer alongside the retinoid X receptor (RXR) [28]. PXR has the ability to recruit numerous co-activators, including members of the p160 co-activator family, such as steroid receptor co-activator 1 (SRC-1), TIF/GRIP (SRC-2), and PPAR co-activator 1a (PGC-1a). PXR undergoes conformational changes upon ligand binding which facilitate its nuclear translocation; as a result of these changes, it recruits co-activators such as SRC-1 and p300/CBP to initiate gene transcription. Our findings demonstrate that autophagy plays a critical role in promoting PXR’s nuclear translocation, suggesting that cellular stress responses may enhance the assembly of the PXR–RXR–coactivator complex. This mechanistic link underscores how autophagy not only supports PXR localization but also potentiates its transcriptional output by stabilizing interactions with RXR and co-activators [29,30].

Moreover, PXR engages in protein–protein interactions with key regulators like p53, Tip60, and p300/CBP, linking it to DNA damage repair and apoptosis pathways. Crosstalk with hypoxia-inducible factors (e.g., HIF-2α) has also been shown to amplify PXR-mediated transcription of drug resistance genes such as CYP3A4, which further complicates therapeutic outcomes in cancer. In addition, various xenobiotics have the ability to activate PXR, which results in the regulation of Phase I and II enzymes, as well as drug transporters. As a result, PXR is recognized for its role in clinical drug–drug interactions in which one medication can enhance the metabolism of another, potentially leading to adverse effects. In addition, several antineoplastic drugs can also stimulate PXR activation, and recent research indicates that this activation may reduce the effectiveness of these medications and contribute to drug resistance during chemotherapy for cancer [31].

Since the majority of CRCs exhibit inherent genomic instability, it is increasingly recognized that therapy-induced genome chaos plays a crucial role in driving drug resistance. Recent studies suggest that anticancer treatments can exacerbate this instability, promoting the emergence of drug-resistant clones through mechanisms such as polyploid giant cancer cells (PGCCs) and chromosomal reorganization. These findings highlight the dynamic interplay between genomic evolution and therapeutic pressure, underscoring the importance of considering genome instability as both a biomarker and a therapeutic challenge in CRC [32,33].

In our experiments, the inhibition of autophagy by HCQ, which alters endosomal transport and autophagosome–lysosome fusion, leads to a reduction in PXR expression and inhibition of PXR translocation from cytoplasm to the nucleus. On the other hand, autophagy induction through inhibition of the PI3K/AKT/mTOR signaling pathway increased the subcellular localization of PXR in the nucleus. Thus, if we combine these data with the reduction in mtKRAS CRC cell viability after the silencing of PXR and treatment with Irinotecan, we conclude that autophagy could act as a protective mechanism through PXR induction for mtKRAS CRC cell lines against this chemotherapeutic agent.

## 5. Conclusions

Overall, these results indicate that autophagy increases the expression of PXR as a resistance mechanism against Irinotecan in mtKRAS CRC cell lines. Autophagy-dependent expression of PXR inhibits apoptotic cell death after treatment with Irinotecan. In addition, autophagy appears to alter the endosomal transport of PXR from the cytoplasm to the nucleus, where PXR acts as a transcription factor. In summary, our data support the hypothesis of the cytoprotective mechanism of autophagy through PXR in mtKRAS CRC. However, it is necessary to establish clinical trials with autophagy inhibitors to investigate whether there will be a benefit in CRC patients with KRAS mutation.

## Data Availability

The data presented in this study are available on request from the corresponding authors. The original Western blot experiments have also been submitted to the journal alongside the manuscript and are available upon request.

## Abbreviations

3-MA	3-Methyladenine
ATCC	American Type Culture Collection
OATP	Anion-transporting polypeptide 2
ABCC	ATP-binding cassette transporter C 2
CRC	Colorectal cancer
DME	Drug-metabolizing enzymes
HCQ	Hydroxychloroquine
MDR1	Multidrug resistance gene 1
mtBRAF	Mutant BRAF
mtKRAS	Mutant KRAS
PXR	Pregnane X receptor
UGTs	UDP-glucuronosyltransferases
UDP	Uridine diphosphate
